# The Georgia Medical Association

**Published:** 1889-05

**Authors:** 


THE GEORGIA MEDICAL ASSOCIATION.—The Georgia Medical Association assembled at Macon on the 18th inst. The Session was held in the Superior Court Room. Dr. W. F. Holt of the City called the Convention to order, and made the address of welcome to the Association. He was responded to by Dr. A. W. Griggs, of West Point. Dr. J. S. Todd as President then delivered his address, the leading feature of which was to show that the medical profession had prolonged the average age of man. The statistics presented in support of this view sustained the position taken. The address was ordered to be published in the public press of Macon. A goodly number of new members were added to the Association, and the attendance was fully up to the average.

A reception was given to the members of the Association by Mrs. H. McHatton, whose husband is a member of the Body..

At the afternoon session of the first day a Committee was apppointed to frame a law for a State Board of Health, to be submitted to the Legislature for adoption. The Committee consists of Drs. J. C. LeHardy, R. B Doster, A. W. Griggs, A. W. Calhoun, Wm. O. Daniel, J. B. Holmes, G. W. Mulligan and Eugene Foster.

Quite a number of Voluntary papers were presented.

The annual Oration was delivered by Dr. J. B. Holmes of Rome.

Dr. Wm. P. Nicolson presented an interesting paper on united fracture of forearm. Operation by drilling and wiring fragments.

At night the Macon Medical Society gave a brilliant reception to the Medical Association, at which there was a sumptuous banquet with dancing, etc.

The officers selected for the ensuing year are as follows: President, J. B. Holmes of Rome; 1st V. President, R. O. Engram, Montezuma; 2nd V. President, P. R. Cortelyou of Marietta; Censor, G. W. Mulligan of Washington; Orator, Dr. W. F. WestMoreland, Jr. of Atlanta.

The place of the next meeting is Brunswick, on the 3rd Wednesday in April, 1890.

