# The Orator-A Mistake

**Published:** 1889-06

**Authors:** 


THE ORATOR—A MISTAKE.—We should have said in our May number that Dr. T. M. Holmes, of Rome, delivered the annual oration at the late meeting of the Georgia Medical Association, and not Dr. J. B. Holmes, as was stated.

